# Integrated Analysis of Disulfidptosis-Related Genes Identifies CD2AP as a Potential Therapeutic Target for Hepatocellular Carcinoma

**DOI:** 10.3390/ijms26094454

**Published:** 2025-05-07

**Authors:** Ning Shang, Jianwei Wang, Zihan Liu, Yake Wang, Di Zhang, Huanfei Liu, Yaqing Zhang, Guifu Dai, Xiaowen Guan

**Affiliations:** 1 School of Life Sciences, Zhengzhou University, Zhengzhou 450001, China; 202011161010127@gs.zzu.edu.cn (N.S.); liuzihan@gs.zzu.edu.cn (Z.L.); mswyk1314@163.com (Y.W.); diudiuzang@163.com (D.Z.); huanfeiliu@163.com (H.L.); zyq13213076252@163.com (Y.Z.); 2 School of Computer and Artificial Intelligence, Zhengzhou University, Zhengzhou 450001, China; wjw725@126.com

**Keywords:** hepatocellular carcinoma, disulfidptosis, prognostic risk model, CD2AP, molecular function

## Abstract

Hepatocellular carcinoma (HCC) is a deadly cancer with limited treatment options for patients at advanced stages. It is urgent to develop reliable prognostic risk models and identify more biomarkers to improve the clinical outcomes of patients with HCC. Disulfidptosis is a newly discovered form of regulated cell death (RCD), and research on the comprehensive roles of disulfidptosis-related genes (DRGs) in HCC prognosis and development remains limited. In this paper, we systematically analyzed the expression levels and prognostic profiles of 26 DRGs in HCC samples from The Cancer Genome Atlas (TCGA) cohort and developed a prognostic risk model using seven hub DRGs. The independent prognostic value of the risk model was further validated in the external cohort. The overall survival of patients with HCC in the low-risk group was significantly longer than that of those in the high-risk group. Subsequently, the protein level of CD2-associated protein (CD2AP) was found to be highly expressed in HCC clinical tissues and associated with the severity of HCC. In vitro experiments demonstrated that the down-regulation of CD2AP attenuated the proliferation, migration, invasion, and epithelial–mesenchymal transition (EMT) abilities of HCC cells. Taken together, our study revealed that the DRG CD2AP may serve as a potential biomarker for HCC and offer support for prognosis prediction of patients with HCC.

## 1. Introduction

Hepatocellular carcinoma (HCC) is one of the most common human malignancies and a leading cause of cancer-related mortality worldwide [1]. With an increasing incidence globally, HCC poses a severe threat to human health. Over the past decade, several advancements, such as radical resection, liver transplantation, and target drugs, have been made in clinical strategies [2]. However, due to the insidious onset, rapid progression, and low early diagnosis rate, most cases of HCC are usually diagnosed at an intermediate or advanced stage and miss the best opportunity for treatment, resulting in a very low 5-year survival rate of less than 20% [3]. Currently, several biomarkers, such as carbohydrate antigen 199, alpha-fetoprotein, glypican-3, and des-gamma-carboxy protein, have been used for diagnosis and risk stratification of HCC [4,5]. Unfortunately, the sensitivity and specificity of these biomarkers are not satisfactory enough. Hence, it is essential to develop credible risk prognosis models and explore more therapeutic targets to improve the diagnosis and treatment of HCC.

Regulated cell death (RCD) refers to the autonomous and orderly death of cells controlled by biomacromolecules [6]. Known RCD forms mainly include apoptosis, necroptosis, pyroptosis, and ferroptosis, which are all closely related to the development of tumors [6]. For example, a previous study suggested the promotive role that necroptosis played in the malignant transformation of cells and cancer progression [7,8]. Pyroptosis was found to inhibit the proliferation and metastasis of multiple cancer cells [9,10]. In addition, ferroptosis-related regulatory factors have been used to estimate the survival of patients with HCC and to distinguish various molecular subtypes of HCC [11,12]. These findings, together, emphasized the importance of RCD in the progression of cancers. Recently, a distinct RCD type from the known RCD forms mentioned above was discovered and termed disulfidptosis [13,14]. Tumor cells highly depend on specific nutrients, such as glucose, glutamine, and glutathione, due to the metabolic reprogramming [15,16,17]. Under the conditions of these nutrients’ starvation, the production of nicotinamide adenine dinucleotide phosphate will become insufficient, resulting in excessive intracellular cystine and the collapse of cytoskeletal proteins [18], ultimately triggering disulfidptosis.

The discovery of disulfidptosis provides a new avenue by which to identify therapeutic targets for cancer treatment by modulating this mode of cell death. A recent study revealed the signature and possible underlying mechanisms of differentially expressed radiotherapy-related disulfidptosis genes in lung adenocarcinoma [19]. The findings of Cong et al. demonstrated that disulfidptosis-related prognostic signature, consisting of six genes (including *CD2AP*), was important for ovarian cancer prognostic assessment, tumor microenvironment modulation, and drug sensitivity prediction [20]. Additionally, several prognostic models of disulfidptosis-related genes (DRGs) for esophageal squamous cell carcinoma, bladder cancer, and diffuse large B-cell lymphoma [21,22,23,24] have also been reported. Based on the existing findings, disulfidptosis was thought to play an important role in cancer progression and prognosis. However, the extent to which disulfidptosis contributes to HCC development and the potential key DRGs have not been sufficiently studied.

As a key organ that controls metabolism and detoxification in the body, enhanced glutathione synthesis is needed to protect the liver from the high-level reactive oxygen species during metabolism processes [25]. Thus, further investigation is essential to elucidate the underlying function and therapeutic applications for DRGs in HCC. Herein, we examined the expression levels and prognostic profiles of DRGs in HCC using The Cancer Genome Atlas (TCGA) cohort and created a DRG risk model designed to predict the prognosis for patients with HCC. The independent prognostic values of the model were further validated in two cohorts and the risk score effectively reflects the stratification and survival of patients. Importantly, we identified *CD2AP* as a new hub DRG for HCC and further investigated the role of CD2AP in HCC cell lines and tissues. Taken together, our results provide direct evidence that *CD2AP* is a potential hub DRG in HCC, expanding the understanding of disulfidptosis in HCC.

## 2. Results

### 2.1. The Landscape of DRGs in HCC

**Figure 1:**
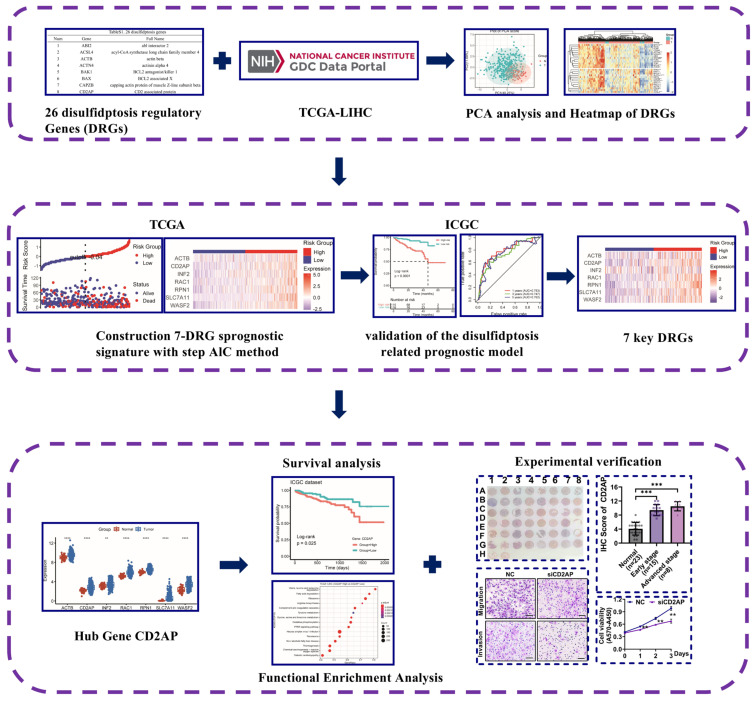
Flowchart of the identification and validation of a disulfidptosis-related prognostic model in HCC and experimental validation of CD2AP. Blue solid line: bioinformatics analysis; blue dashed line: in vitro assays; 26 disulfidptosis regulatory Genes were listed in Table S1; DRGs, disulfidptosis-related genes; HCC, hepatocellular carcinoma; ICGC, International Cancer Genome Consortium; PCA, principal component analysis; TCGA-LIHC, The Cancer Genome Atlas Liver Hepatocellular Carcinoma. * *p* < 0.05, ** *p* < 0.01, *** *p* < 0.001, **** *p* < 0.0001.

**Figure 2:**
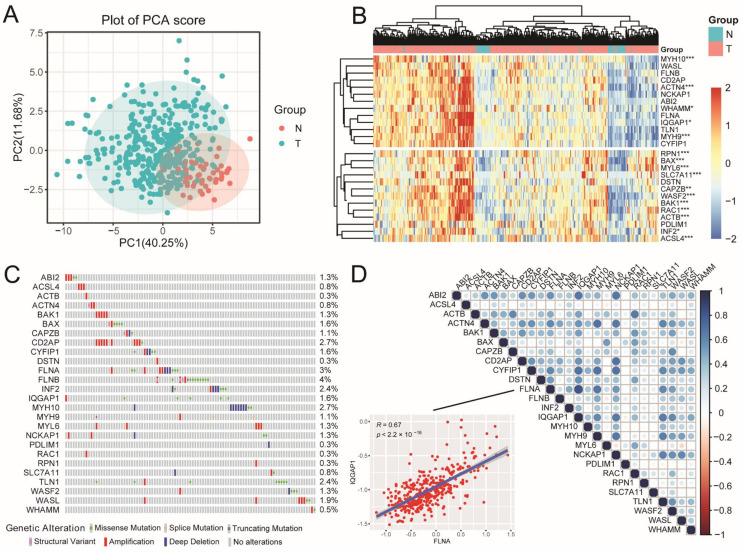
Landscape of DRGs in HCC. (**A**) PCA for DRGs in normal and tumor samples from TCGA cohort. (**B**) Heatmap showing the relative expression of 26 DRGs between the normal (N, brilliant blue) and tumor tissues (T, red) from TCGA cohort. *p*-values are shown as follows: (**C**) Genetic alteration of DRGs in HCC. (**D**) Pearson’s correlation analysis among the DRGs. Red plot: negative correlation; blue plot: positive correlation. The depth of the colors reflects the strength of the correlation. * *p* < 0.05, ** *p* < 0.01, *** *p* < 0.001.

According to the flow chart of the overall experimental design (Figure 1), we first collated a list of 26 DRGs from the reported articles [26] (Table S1). Next, differential expression analysis of these DRGs in normal and tumor tissues was performed using The Cancer Genome Atlas Liver Hepatocellular Carcinoma (TCGA-LIHC) cohort. The results showed that the expression of all the DRGs was significantly distinguished between normal and tumor tissues (Figure 2A), and most of the DRGs were highly expressed in tumor tissues (Figure 2B, *p* < 0.05). Furthermore, the Gene Ontology (GO) and Kyoto Encyclopedia of Genes and Genome (KEGG) analysis displayed that DRGs were not only enriched in filament-based processes, the actin cytoskeleton, and adherens junctions, which were important biological processes for disulfidptosis; they were also enriched in cancer-related pathways such as tight junction, the invasion of epithelial cells, and focal adhesion (Figure S1). Another genetic alteration analysis revealed higher mutation frequency (Figure 2C) in *FLNB* (4%), *FLNA* (3%), *CD2AP* (2.7%), and *MYH10* (2.7%). Simultaneously, significant correlations were observed among the DRGs (Figure 2D); for example, *FLNA* was positively correlated with *IQGAP1* (*R* = 0.67, *p* < 2.2 × 10^−16^), suggesting potential coordinated functions between these DRGs in tumor regulation. These findings clarify the expression and mutation profiles of these 26 DRGs in HCC.

### 2.2. Construction and Validation of the Disulfidptosis-Related Prognostic Model

**Figure 3:**
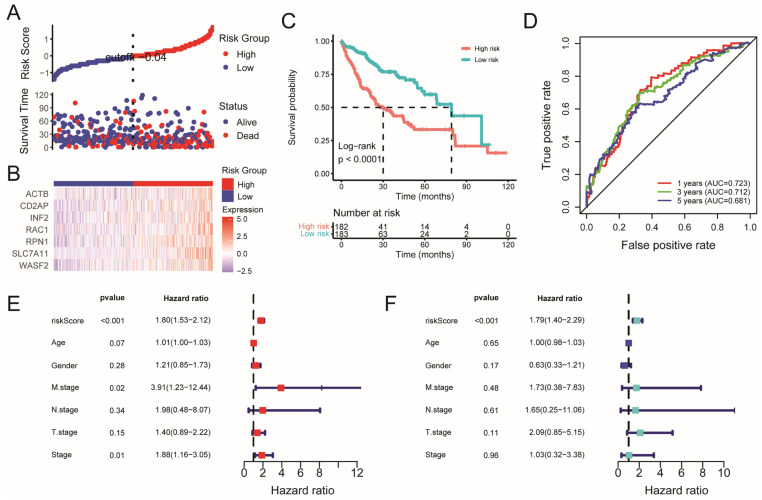
Construction of the disulfidptosis-related prognostic model. (**A**) Surveying the risk model in the TCGA cohort: distribution of risk score and survival status of prognostic signatures in patients with HCC between the high- and low-risk groups. (**B**) Heatmap showing the distribution expression of the seven DRGs. (**C**) KM survival curve for patients with HCC in high- and low-risk groups. (**D**) The predictive efficiency of the risk score demonstrating the ROC curves in the TCGA cohort with 1, 3, and 5 years. Univariate (**E**) and multivariate (**F**) Cox regression analysis of risk score and clinical characteristics. AUC, areas under the curve; KM survival curve, Kaplan–Meier survival curve, ROC, receiver operating characteristic.

To investigate the potential of DRGs on the prognosis of HCC, univariate Cox regression analysis was performed for the primary screening (Figure S2A), and we obtained 13 DRGs whose hazard ratios were significantly greater than 1 (*p* < 0.05). Furthermore, the stepAIC algorithm was performed to establish an optimal prognostic risk model based on seven DRGs, namely, *ACTB, CD2AP, INF2*, *RAC1*, *RPN1*, *SLC7A11*, and *WASF2*. The risk score was calculated using the following equation: risk score = (0.35587 × exp*SLC7A11*) + (0.39395 × exp*RAC1*) + (0.58464 × exp*RPN1*) + (0.24345 × exp*WASF2*) + (0.26576 × exp*INF2*) − (0.42347 × exp*ACTB*) + (0.03096 × exp*CD2AP*). Subsequently, the patients with HCC in the TCGA cohort were assigned into high- and low-risk groups according to the median risk score. As shown in Figure 3A,B, the prognosis of patients in the high-risk group was characterized by a higher mortality rate and reduced survival time compared to those in the low-risk group, and the expression of all the seven DRGs was upregulated in the high-risk group. Kaplan–Meier (KM) analysis demonstrated a statistically significant difference in survival rates between the low- and high-risk groups (*p* < 0.001) (Figure 3C), and the high-risk group patients had a worse overall survival rate. Additionally, time-dependent receiver operating characteristic (ROC) analysis showed that the areas under the curve (AUC) of the ROC were 0.723 for 1-year, 0.712 for 3-year, and 0.681 for 5-year overall survival (Figure 3D), which suggests a high accuracy prediction of the prognostic risk model. Next, to further evaluate the clinical utility of the risk score, we integrated clinical data along with additional variables including risk score, age, gender, M stage, N stage, and T stage. The results from both univariable Cox regression and multivariable Cox regression analysis indicated that the risk score could serve as an independent prognostic factor within the TCGA cohort (Figure 3E,F) (*p* < 0.001). We also established a nomogram to quantitatively predict the 1-, 3-, and 5-year survival probabilities for patients with HCC, which exhibited excellent predictive performance of the constructed prognostic model (Figure S2B). Furthermore, the KEGG analysis of differentially expressed genes (DEGs) between the high- and low-risk groups showed that the DEGs were significantly enriched in a variety of metabolic pathways, such as retinol metabolism, glycolysis/gluconeogenesis, and tyrosine metabolism (Figure S3A,B), demonstrating the potential mechanism of the risk signature related to the metabolism process.

**Figure 4:**
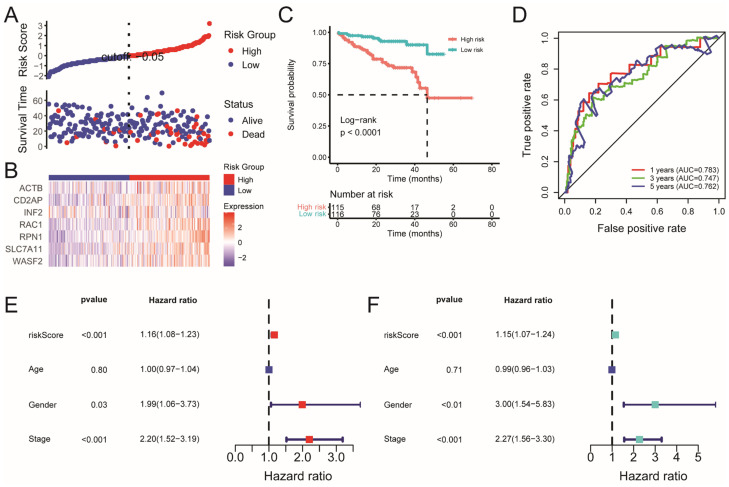
External validation of the disulfidptosis-related prognostic model. (**A**) Surveying the risk model in the ICGC cohort: distribution of risk score and survival status of prognostic DRGs in patients with HCC between the two risk groups. (**B**) Heatmap showing the distribution expression of the seven prognostic DRGs. (**C**) KM survival curves for patients with HCC in high-and low-risk groups. (**D**) The predictive efficiency of the risk score demonstrating the ROC curves in ICGC cohort. Univariate (**E**) and multivariate (**F**) Cox regression analysis of risk score and clinical characteristics.

We further validated the prognostic model in an external validation set consisting of 231 patients with HCC from the International Cancer Genome Consortium (ICGC) cohort. patients with HCC in the high-risk group still had a higher mortality rate and shorter survival time than those in the low-risk group (Figure 4A,B). Moreover, KM analysis also revealed a significant difference in survival rates between the low- and high-risk groups (*p* < 0.001) (Figure 4C). The ROC curve analysis of the ICGC dataset demonstrated that the constructed prognostic model exhibited strong predictive efficacy (AUC = 0.783 for 1-year, 0.747 for 3-year, and 0.762 for 5-year survival) (Figure 4D). More importantly, the risk score was also identified as independent prognostic factors in the ICGC cohort, as evidenced by both univariable and multivariable Cox regression analysis (Figure 4E,F). Taken together, these results indicate that the prognostic risk model can effectively stratify the high- and low-risk groups and predict the survival of patients with HCC.

### 2.3. Identifying CD2AP as a New Hub DRG for HCC

**Figure 5:**
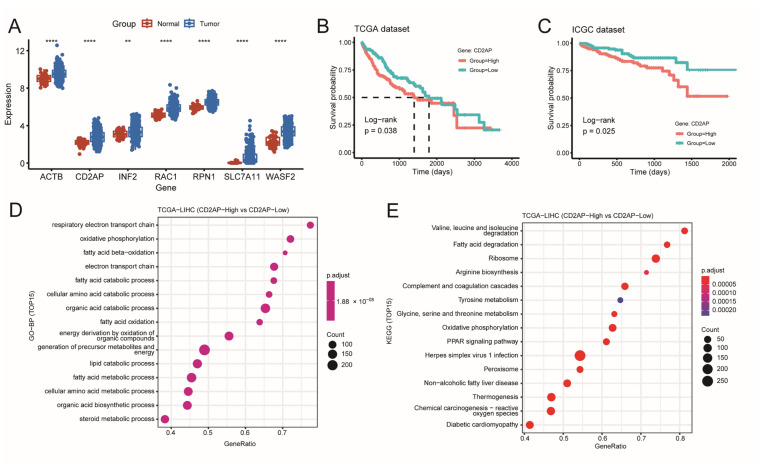
Identifying *CD2AP* as a new hub DRG for HCC. (**A**) Gene expression distributions for seven risk DRGs are presented as box plots, where tumor specimens (blue) are compared against matched normal tissues (red). (**B**,**C**) OS curves for patients with HCC with high and low *CD2AP* expression in TCGA dataset (**B**) and ICGC dataset (**C**). (**D**,**E**) GSEA analysis of the DEGs between high- and low-expression *CD2AP* groups. CD2AP: CD2-associated protein; GO-BP, gene ontology biological process; GSEA, Gene Set Enrichment Analysis; KEGG, Kyoto Encyclopedia of Genes and Genome. ** *p <* 0.01, **** *p <* 0.0001.

We analyzed the expression levels of the seven DRGs in both tumor and normal tissues from the TCGA cohort. The box plots showed that these seven genes exhibited higher expression levels in HCC tissues compared to normal tissues (Figure 5A) (*p* < 0.01). Subsequent KM analysis showed that the low expression levels of *SLC7A11*, *RAC1*, *RPN1*, *WASF2,* and *CD2AP* were associated with significantly longer overall survival (OS) compared to high expression groups in both the TCGA (Figure S4A and Figure 5B) (*p* < 0.05) and ICGC datasets (Figure S4B and Figure 5C) (*p* < 0.05), while there was no difference in OS between patients with low- and high-expression of *ACTB* and *INF2* in the ICGC cohort. Notably, as previously mentioned, *SLC7A11*, *RAC1*, *RPN1,* and *WASF2* play oncogenic roles in the progression of HCC. For example, RPN1 was reported to be a prognosis gene for HCC and was associated with poorer survival and metastasis in HCC [27]. WASF2 has been reported to be overexpressed and hypomethylated in HCC and correlates with patient prognosis [28]. RAC1 plays an important role in cell invasion/metastasis of HCC [29]. SLC7A11 was found to be overexpressed in various cancer types, including HCC, and plays a role in tumor promotion [30]. However, the role of CD2AP in HCC remains poorly understood, which drives us to further explore the potential biological function of CD2AP in HCC progression, and we obtained a total of 1323 DEGs between the high- and low-expression CD2AP groups in TCGA-LIHC cohort and further Gene Set Enrichment Analysis (GSEA) and KEGG analysis revealed that these DEGs were significantly enriched in pathways related to fatty acid oxidation, degradation, catabolic process, and various metabolic pathways, including retinol metabolism and tyrosine metabolism, which are closely related to the process of disulfidptosis (Figure 5D,E and Figure S5A,B) and consistent with the analysis results of DEGs between high- and low-risk group in Figure S3B. Overall, these results indicate that *CD2AP* may be an important factor in HCC progression.

### 2.4. CD2AP Is Highly Expressed in HCC Cells and Tissues

**Figure 6:**
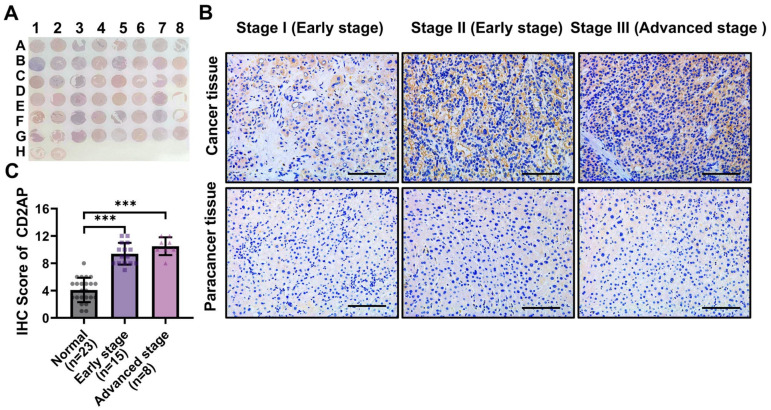
CD2AP is highly expressed in HCC clinical tissues. (**A**) Representative HCC tissue microarray: the units in lines 1, 3, 5, and 7 represent tumor tissues, and the units in lines 2, 4, 6, and 8 represent the para-carcinoma tissues. (**B**) IHC staining for CD2AP protein expression in three representatives of the paired tumor tissues and para-carcinoma tissues derived from clinical patients with HCC. Scale bars: 100 μm. (**C**) The IHC score for CD2AP in HCC tissues and the para-carcinoma tissues (early stage: stage I and II, n = 15; advanced stage: stage III, n = 8). Data are represented as the mean ± SD. Mann–Whitney U test was used to calculate *p*-values. *** *p* < 0.001 versus the corresponding control. IHC: immunohistochemistry.

To explore the role of CD2AP in HCC, the expression of CD2AP was further validated through an HCC tissue microarray, and the results demonstrated that the expression of CD2AP was markedly increased in the tumor tissues compared with the para-carcinoma tissues and positively correlated with the severity of HCC (Figure 6A–C). These results demonstrate that CD2AP is significantly elevated in HCC tissues and cells and might play an important role in regulating HCC progression.

### 2.5. CD2AP Knockdown Restrains the Proliferation, Migration, and Invasion Abilities of HCC Cells

**Figure 7:**
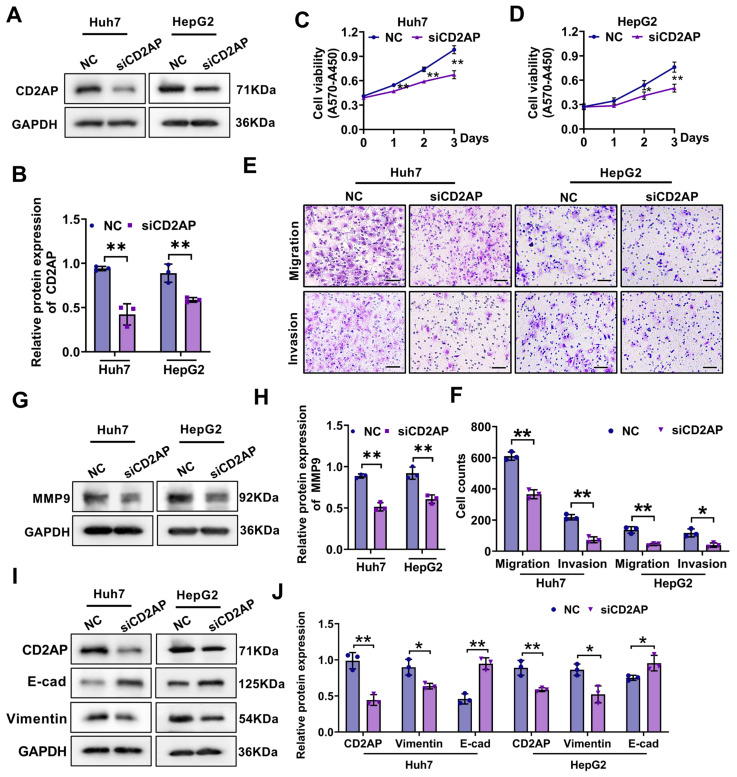
Inhibition of CD2AP restrains proliferation, migration, and invasion capacities of HCC cells. (**A**) The protein levels of CD2AP in the CD2AP-knockdown and the corresponding control cells. (**B**) Densitometric quantitation of CD2AP in the indicated cells. (**C**,**D**) Growth curves showing the viabilities of cells after the knockdown of CD2AP in Huh7 and HepG2 cells. (**E**,**F**) Transwell assay was used to evaluate the migration and invasion of Huh7 and HepG2 cells after the knockdown of CD2AP (Scale bars, 50 µm). (**G**) The protein levels of MMP9 in the CD2AP-knockdown cells and the corresponding control cells. (**H**) Densitometric quantitation of MMP9 in the indicated cells. (**I**) The protein levels of E-cadherin and vimentin in the CD2AP-knockdown cells and the corresponding control cells. (**J**) Densitometric quantitation of E-cadherin and vimentin and CD2AP in the indicated cells. Data are shown as the mean ± SD from three independent experiments. Student’s *t*-test was used to calculate the *p*-values. * *p* < 0.05, ** *p* < 0.01 versus the corresponding control. MMP-9, matrix metalloproteinase-9; E-cad: E-cadherin.

To further investigate the biological function of CD2AP in HCC, we employed small interfering RNA (siRNA) tool to knock down the CD2AP expression, and the siRNAs successfully silenced CD2AP protein expression in both Huh7 and HepG2 cells efficiency (Figure 7A,B). It was found that the suppression of CD2AP expression significantly attenuated the proliferative capacity of Huh7 and HepG2 cells (Figure 7C,D). Furthermore, Transwell analysis revealed an obvious reduction in the number of migrated and invasive cells in CD2AP-knockdown cells compared to the corresponding controls (Figure 7E,F). Since studies reported that matrix metalloproteinase-9 (MMP9) was crucial to the progression of cell migration and invasion [31], the regulatory effect of CD2AP on MMP9 was investigated, and the results demonstrated that CD2AP knockdown remarkably suppressed the protein expression levels of MMP9 in Huh7 and HepG2 cells (Figure 7G,H). Epithelial–mesenchymal transition (EMT) has been widely demonstrated to be associated with various cellular processes such as invasion, metastasis, and immunosuppression [32]. A recent study has reported that CD2AP/TKS4 complexes could regulate migration and EMT-related pathways in colon cancer cells [33]. Based on these findings, in order to further explore whether CD2AP affect the EMT process, two EMT-related genes, E-cadherin and vimentin, were detected using Western blot analysis after the knockdown of CD2AP. Notably, the inhibition of CD2AP expression significantly increased the expression of E-cadherin, while it decreased the expression of vimentin (Figure 7I,J). Altogether, these findings reveal the important role of CD2AP in regulating the EMT process. Collectively, these results suggest that the inhibition of CD2AP substantially suppresses the proliferation, migration, and invasion capacities of HCC cells, indicating a potential pro-tumorigenic role in HCC development.

## 3. Discussion

RCD forms, caused by dysfunctional and deregulated cell signaling, play a central role in all aspects of cellular biological processes. For example, apoptosis is the best-studied form of RCD and can be the primary target for most cancer treatment strategies [6,34,35]. Other RCD forms, including necroptosis, autophagy, ferroptosis, and cuproptosis, have also been recognized as typical markers for the diagnosis and prognosis of various cancers [6,36,37]. Recently, disulfidptosis has emerged as a novel RCD mechanism, offering a new avenue for cancer therapy [14]. A comprehensive understanding for the prognostic characteristics of disulfidptosis in HCC, along with the identification and function of hub DRGs is necessary.

Several studies have demonstrated the potential of DRGs for constructing prognostic models in several tumors, such as gastric cancer and kidney renal clear cell carcinoma [14]. In the current study, we found that most DRGs were upregulated in tumor tissues from patients with HCC in the TCGA cohort, which were consistent with the results of previous studies [38,39,40]. Furthermore, a prognostic risk model was constructed based on seven DRGs (*CD2AP*, *ACTB*, *INF2*, *WASF2*, *RPN1*, *RAC1*, and *SLC7A11*), and combined with univariate and multivariate regression prognosis models, we found that the model could predict the prognosis of patients well. Additionally, KEGG analysis showed that the DEGs between high- and low-risk groups were significantly enriched in pathways related to metabolism, such as xenobiotics metabolism, retinol metabolism, and glycolysis/gluconeogenesis. It is widely accepted that metabolic reprogramming is a critical hallmark of cancer progression [15,16,17]. During tumorigenesis, cancer cells often reprogram themetabolic pathways that control glycolysis, the tricarboxylic acid cycle (TCA), and glutaminolysis. Retinol and active glycolysis commonly contribute to cancer development [15,41], such as the malignant progression, therapy resistance, and immune tolerance of various cancers [17], indicating that retinol metabolism and glycolysis may be the main metabolic driving forces of high-risk progression. More importantly, in our study, the OS of patients with HCC in the low-risk group was significantly longer than that in the high-risk group, which provides evidence affirming the efficacy of the prognostic risk model in accurately predicting the survival of patients with HCC.

Notably, among these seven DRGs, it was found that five genes, namely, *RAC1*, *RPN1*, *WASF2*, *SLC7A11,* and *CD2AP*, produced significant differences in both the TCGA and ICGC cohorts. It was reported that *RPN1* was a prognosis gene for HCC and was associated with poorer survival and metastasis in HCC [27]. WASF2 has been reported to serve as a potential therapeutic target for HCC [28]. Moreover, given the function of signal transduction from the external cell to the actin cytoskeleton, RAC1 plays an important role in cell invasion/metastasis of several cancers [29]. Another important gene in disulfidptosis, SLC7A11 was found to be overexpressed in various cancer types, including HCC, and plays a role in tumor promotion [30]. As for CD2AP, researchers have identified it as a diagnostic and prognostic biomarker for patients with renal clear cell carcinoma [42]. These findings together showed that these five key DRGs played important roles in the progression of HCC or other cancers. Notably, unlike the other four DRGs, the role of CD2AP in HCC development has not been explored, indicating that CD2AP may represent a new hub DRG associated with the development of HCC. Additionally, GSEA and KEGG analysis revealed that the DEGs between high- and low-expressed CD2AP groups were significantly enriched in metabolism pathways including retinol metabolism, tyrosine metabolism, fatty acid oxidation, and catabolic process, which is consistent with the pathway analysis of DEGs between high- and low-risk groups. In particular, FA metabolism plays a critical role as a source of energy, facilitates microenvironmental adaptation, and mediates cellular signaling during the initiation and progression of HCC [43,44], further providing evidence for the close correlation between CD2AP and the progression of HCC.

More surprisingly, in vitro experiments showed that CD2AP was highly expressed in both HCC tissues and cells, and knockdown of CD2AP inhibited the capabilities of proliferation, migration, and invasion in Huh7 and HepG2 cells, which is consistent with the oncogenic role of CD2AP in other cancers [45,46]. It is worth noting that recent research demonstrated the critical role of CD2AP in dynamic actin remodeling and membrane trafficking, underscoring its significance in the formation of epithelial cell junctions [47]. Another study revealed that CD2AP and TKS4 could form a scaffolding protein complex to regulate migration and EMT-related pathways in colon cancer cells [33]. As is accepted widely, EMT is a biological process in which the expression levels of cell adhesion and cytoskeleton molecules were altered to make epithelial cells transition into mesenchymal-like cells and gain migratory and invasive properties [48], unveiling the importance of the EMT process for cancer cell invasion and metastasis. Similarly, our study showed that the protein level of the EMT-related marker E-cadherin was significantly increased, while the expression of vimentin was reduced after the knockdown of CD2AP, suggesting that CD2AP plays a crucial role in the process of EMT during HCC development. Intriguingly, these findings may hold potential for clinical implications. The risk model can be used for reliable stratification of patients with HCC, facilitating personalized management strategies based on individual molecular profiles. In addition, siRNA/shRNA-mediated CD2AP silencing could also potentially inhibit tumor metastasis, building on the existing delivery platforms from other oncogene-targeting trials. But these proposed therapeutic strategies will require further in-depth investigation and validation.

Inevitably, there are some limitations in our study. First, our work provided substantial evidence for the seven-DRG risk model being able to effectively predict the survival of patients with HCC. Nonetheless, it is still necessary to validate the risk model within several cohorts from real clinical tissues. Second, the study on the effect of CD2AP on HCC progression is not fully complete; additional investigations are required. Further in vivo and in vitro experiments, such as CD2AP knockout mouse models and 3D HCC cell models, are necessary in order to comprehensively explore the biological significance of CD2AP.

In conclusion, our study successfully develops a prognostic risk model based on seven DRGs for HCC and identifies a novel hub DRG, CD2AP, which affects proliferation, invasion, migration, and EMT process in HCC progression. These advancements provide a reliable prognostic model for predicting the outcomes of patients with HCC and present a promising therapeutic target for HCC treatment.

## 4. Materials and Methods

### 4.1. Data Acquisition

The gene expression data and corresponding clinical information of hepatocellular carcinoma (HCC) samples were obtained from The Cancer Genome Atlas (TCGA) (https://portal.gdc.cancer.gov/) (accessed on 1 December 2023) and Cancer Genome Consortium (ICGC)(https://icgc.org/) databases (accessed on 1 December 2023). For all the RNA-seq data, normalization and log2(x + 1) transformation were performed. A total of 26 disulfidptosis-related genes (DRGs) were retrieved from the most recent literature (listed in Table S1). The STRING (https://string-db.org/) (accessed on 1 February 2024) and Cytoscape (https://cytoscape.org/) tools (accessed on 1 February 2024) were used to construct the selected genes’ protein–protein interaction (PPI) networks.

### 4.2. Construction of Risk Prognostic Signature

To investigate the prognostic value of the selected genes, we first used univariate Cox regression analysis of the DRGs and then applied the stepAIC algorithm to construct a prognostic risk model. Based on the prognostic potential and the regression coefficients, seven genes were identified, and the risk signature was finally developed. The risk score of every HCC patient was calculated according to the following formula:

$$Riskscore=\sum_{i=1}^{n}C{oef}_{gene}\times {exp}_{gene}$$

with Coef indicating the coefficient and exp indicating the expression level of genes. Patients with a risk score above the median value were classified as the high-risk group, while the remaining patients with HCC were identified as the low-risk group.

### 4.3. Survival Analysis

A Kaplan–Meier (KM) survival analysis of the gene signature from the TCGA and ICGC datasets was performed, and comparisons among different groups were made using the log-rank test. For Kaplan–Meier curves, *p*-values and hazard ratio (HR) with a 95% confidence interval (CI) were generated using log-rank tests and univariate Cox proportional hazards regression. HR represents the hazard ratio of the low-expression sample relative to the high-expression sample. An HR greater than 1 indicates that the gene is a risk factor, while an HR less than 1 indicates that the gene is a protective factor. The predictive efficiency of genes for 1-, 3-, and 5-year survival was estimated using receiver operating characteristic (ROC) curves generated by the “timeROC” package. Univariate and multivariate Cox regression analyses were performed using clinical features (age, gender, T, N, M, and tumor stage) and risk scores to identify the appropriate terms for building the nomogram. The forest was used to show the *p*-value, HR, and 95% CI of each variable through the “forest plot” R package.

### 4.4. Functional Enrichment Analysis

The data were analyzed via functional enrichment to further clarify the functions underlying potential targets. To better understand the carcinogenesis of mRNA, the “Cluster Profiler” package in R was employed to analyze the Gene Ontology (GO) functions of potential targets and enrich the Kyoto Encyclopedia of Genes and Genomes (KEGG) pathways. The differentially expressed genes (DEGs) were identified in the high-risk and low-risk groups, and the DEGs between the high CD2AP expression group and the low CD2AP expression group were calculated using the limma package. These were then screened with an FDR < 0.05 and |Fold change| > 1.5 to identify significant differences. The results of the GO and KEGG analysis were visualized using the “ggplot2” package.

### 4.5. Human Liver Specimens

A human liver tissue chip (Cat# HLivHCC050PG01, Outdo Biotech Co., Ltd., Shanghai, China), a fresh-frozen liver tissue microarray, which contained HCC tissues and the corresponding para-cancerous tissues from 25 patients, was utilized in this study. Among the patients, 2 patients were diagnosed with intrahepatic cholangiocarcinoma and the rest with hepatocellular carcinoma.

### 4.6. Immunohistochemistry (IHC) Staining

The human liver tissue chip was deparaffinized with xylene and graded ethanol and then washed in water for 5 min. Following this, the chip was incubated in 3% hydrogen peroxide for 15 min to remove peroxidase and soaked in EDTA alkaline repair solution (pH = 9.0) at 95–100 °C for 15 min to perform antigen repair. The slide was then blocked with 2% BSA for 1 h at room temperature and incubated with the primary antibody (CD2AP; 1:200; Proteintech, Wuhan, China) overnight at 4 °C. After incubation with the secondary antibody (1:300, Thermo Fisher Scientific, Waltham, MA, USA) for 1 h, the chip was stained with 3, 3′-diaminobenzidine. The IHC score was determined according to the intensity and the area of the positively stained region. The evaluation standard for the percentage score is as follows: negative stained, percentage score 0; <20% of positively stained region, percentage score 1; 20–50% of positively stained region, percentage score 2; and >50% of positively stained region, percentage score 3. The intensity score is based on the following guidelines: 0, indicating negative staining; 1, indicating weak staining; 2, indicating moderate staining; and 3, indicating strong staining. The resulting score was calculated by multiplying the percentage score and the intensity score.

### 4.7. Cell Culture

Huh7 cells were obtained from the Cell Resource Center, Institute of Basic Medical Sciences, Chinese Academy of Medical Sciences (Beijing, China). HepG2 cells were purchased from the Kunming Cell Bank, Committee on Type Culture Collection, Chinese Academy of Sciences (Kunming, China). Cells were cultured in high-glucose DMEM (Gibco, Thermo Fisher Scientific, Waltham, MA, USA) containing 10% fetal bovine serum (FBS; Yeasen, Shanghai, China), 100 U/mL of penicillin, and 100 μg/mL of streptomycin. All cells were placed in an incubator at a constant temperature of 37 °C and carbon dioxide concentration of 5%.

### 4.8. Cell Viability

Huh7 and HepG2 cells were plated into a 6-well plate and then transfected with siRNA (NC or siCD2AP). After 24 h of transfection, 5000 cells were transferred to 96-well plates and incubated for another 24, 36, or 48 h. Cell viability was then measured using the MTT assay. At every designated time interval, MTT was added into the cell cultures at a final concentration of 0.5 mg/mL at 37 °C and incubated for 4 h. Subsequently, the supernatant was replaced with 150 microliters of DMSO, followed by measuring the absorbance at 570 nm and 450 nm utilizing a microplate reader (Thermo Fisher Scientific, Waltham, MA, USA). The cell vitality was calculated as A_570_ minus A_450_.

### 4.9. Migration and Invasion Assay

The migration and invasion of Huh7 and HepG2 cells were determined in a transwell chamber (Corning, NY, USA) containing a polycarbonate filter with a pore size of 8 μm. For the migration assay, cells were collected and resuspended in serum-free DMEM medium after being transfected with NC or siCD2AP for 12 h. A total of 4 × 10^5^ (Huh7) or 8 × 10^5^ (HepG2) cells were seeded into the upper compartment of each chamber. The lower compartment contained 0.6 mL high-glucose DMEM medium with 10% FBS. After incubation for 48 h at 37 °C, the filters were removed and fixed with 4% paraformaldehyde. Cells on the lower surface of the filter were stained with 1% crystal violet for 30 min. The stained cells were imaged using an upright microscope at a magnification of 100× and analyzed using the software GraphPad Prism (version 9.0; GraphPad Software, LLC, San Diego, CA, USA). For the invasion assay, the Matrigel (BD Biosciences, San Jose, CA, USA) was diluted with high-glucose DMEM medium at a ratio of 1:8. Then, 60 μL of the diluted Matrigel was added to the transwell chamber and placed in a 37 °C incubator for 3 h to solidify. The rest of the experiments were conducted as described in the migration assay.

### 4.10. Western Blot Analysis

Protein samples were obtained by lysing cells with RIPA lysis buffer (Beyotime, Shanghai, China). Equal amounts of protein were transferred onto nitrocellulose membranes (Pall Corporation, Port Washington, NY, USA) after separation via 10% SDS—PAGE. Subsequently, the membranes were blocked with 5% skim milk for 1 h and incubated overnight with diluted primary antibodies against CD2AP (1:1000; Proteintech, Wuhan, China), MMP-9 (1:2000; HUABIO, Zhejiang, China), E-cadherin (1:2500; Proteintech, Wuhan, China), and vimentin (1:1000; HUABIO, Zhejiang, China). After incubation with the secondary antibody (1:4000; Thermo Fisher Scientific, Waltham, MA, USA) for 1 h, the target bands were visualized using a chemiluminescence imager (C300, Azure Biosystems, Dublin, CA, USA) and analyzed with Image J software (Image J, version 1.51j8, NIH, Bethesda, MD, USA).

### 4.11. Statistical Analysis

The R software package “ggplot2” was used to draw boxplot, while the “heatmap” package was utilized to create heatmaps. The predictive efficiency of genes for overall survival (OS) was estimated using ROC curves generated by the “timeROC” package. The statistical difference between the two groups was compared through the Wilcox test. For the biological experiments, data analyses were conducted using SPSS software (version 25.0; IBM, Armonk, NY, USA) and GraphPad Prism (version 9.0; GraphPad Software, LLC, San Diego, CA, USA). Student’s *t* test or the Mann—Whitney U test were used to compare values between the two groups. The data are presented as the mean ± standard deviation (SD) from three biological independent experiments. *p* < 0.05 was considered statistically significant (* *p* < 0.05; ** *p*  < 0 .01, *** *p*  < 0 .001).

## Data Availability

Publicly available datasets were analyzed in this study. These data can be found in TCGA: https://portal.gdc.cancer.gov/ (tumor id: LIHC) (accessed on 1 December 2023) and https://dcc.icgc.org/ (tumor id: LIRI-JP) (accessed on 1 December 2023).

## Supplementary Materials

The following supporting information can be downloaded at: https://www.mdpi.com/article/10.3390/ijms26094454/s1.

## References

[1] Jemal A., Ward E.M., Johnson C.J., Cronin K.A., Ma J., Ryerson A.B., Mariotto A., Lake A.J., Wilson R., Sherman R.L. (2017). Annual Report to the Nation on the Status of Cancer, 1975–2014, Featuring Survival. JNCI-J. Natl. Cancer Inst..

[2] Fu Y.J., Liu S.S., Zeng S., Shen H. (2019). From bench to bed: The tumor immune microenvironment and current immunotherapeutic strategies for hepatocellular carcinoma. J. Exp. Clin. Cancer Res..

[3] Allemani C., Weir H.K., Carreira H., Harewood R., Spika D., Wang X.S., Bannon F., Ahn J.V., Johnson C.J., Bonaventure A. (2015). Global surveillance of cancer survival 1995–2009: Analysis of individual data for 25,676,887 patients from 279 population-based registries in 67 countries (CONCORD-2). Lancet.

[4] Toyoda H., Kumada T., Tada T., Sone Y., Kaneoka Y., Maeda A. (2015). Tumor Markers for Hepatocellular Carcinoma: Simple and Significant Predictors of Outcome in Patients with HCC. Liver Cancer.

[5] Li H., Qu K.Z., Ner R.M., Sferruzza A.D., Bender R.A. (2007). Preliminary study of three tumor markers for hepatocellular carcinoma: AFP, AFP-L3, and Glypican-3. J. Clin. Oncol..

[6] Peng F., Liao M., Qin R., Zhu S., Peng C., Fu L., Chen Y., Han B. (2022). Regulated cell death (RCD) in cancer: Key pathways and targeted therapies. Signal Transduct. Target. Ther..

[7] Seehawer M., Heinzmann F., D’Artista L., Harbig J., Roux P.F., Hoenicke L., Dang H., Klotz S., Robinson L., Doré G. (2018). Necroptosis microenvironment directs lineage commitment in liver cancer. Nature.

[8] Yang Y.C., Jiang Q., Yang K.P., Wang L., Sethi G., Ma Z. (2024). Extracellular vesicle-mediated ferroptosis, pyroptosis, and necroptosis: Potential clinical applications in cancer therapy. Cell Death Discov..

[9] Fang Y., Tian S.W., Pan Y.T., Li W., Wang Q.M., Tang Y., Yu T., Wu X., Shi Y.K., Ma P. (2020). Pyroptosis: A new frontier in cancer. Biomed. Pharmacother..

[10] Yu J.H., Li S., Qi J., Chen Z.L., Wu Y.H., Guo J., Wang K., Sun X.J., Zheng J.B. (2019). Cleavage of GSDME by caspase-3 determines lobaplatin-induced pyroptosis in colon cancer cells. Cell Death Dis..

[11] Long S.C., Chen Y.Q., Wang Y., Yao Y.B., Xiao S., Fu K. (2023). Identification of Ferroptosis-related molecular model and immune subtypes of hepatocellular carcinoma for individual therapy. Cancer Med..

[12] Zhang W.J., Yao S.M., Huang H., Zhou H., Zhou H.M., Wei Q.S., Bian T.T., Sun H., Li X.L., Zhang J.G. (2021). Molecular subtypes based on ferroptosis-related genes and tumor microenvironment infiltration characterization in lung adenocarcinoma. OncoImmunology.

[13] Liu X.G., Zhuang L., Gan B.Y. (2024). Disulfidptosis: Disulfide stress-induced cell death. Trends Cell Biol..

[14] Zheng P.J., Zhou C.T., Ding Y.M., Duan S.W. (2023). Disulfidptosis: A new target for metabolic cancer therapy. J. Exp. Clin. Cancer Res..

[15] Qu H., Liu J., Zhang D., Xie R., Wang L., Hong J. (2023). Glycolysis in Chronic Liver Diseases: Mechanistic Insights and Therapeutic Opportunities. Cells.

[16] Shuvalov O., Daks A., Fedorova O., Petukhov A., Barlev N. (2021). Linking Metabolic Reprogramming, Plasticity and Tumor Progression. Cancers.

[17] Hu B., Yu M.C., Ma X.L., Sun J.L., Liu C.L., Wang C.Y., Wu S.Y., Fu P.Y., Yang Z., He Y.G. (2022). IFNa Potentiates Anti-PD-1 Efficacy by Remodeling Glucose Metabolism in the Hepatocellular Carcinoma Microenvironment. Cancer Discov..

[18] Koppula P., Zhuang L., Gan B.Y. (2021). Cystine transporter SLC7A11/xCT in cancer: Ferroptosis, nutrient dependency, and cancer therapy. Protein Cell.

[19] Pan X.X., Qian H.Y., Sun Z.A., Yi Q., Liu Y., Lan G.Z., Chen J., Wang G.R. (2024). Investigating the role of disulfidptosis related genes in radiotherapy resistance of lung adenocarcinoma. Front. Med..

[20] Cong Y.Y., Cai G.Y., Ding C.C., Zhang H., Chen J.P., Luo S.W., Liu J.H. (2024). Disulfidptosis-related signature elucidates the prognostic, immunologic, and therapeutic characteristics in ovarian cancer. Front. Genet..

[21] Zhao S.Y., Wang L.Y., Ding W., Ye B.C., Cheng C., Shao J.F., Liu J.H., Zhou H.Y. (2023). Crosstalk of disulfidptosis-related subtypes, establishment of a prognostic signature and immune infiltration characteristics in bladder cancer based on a machine learning survival framework. Front. Endocrinol..

[22] Liu F.X., Yuan D.L., Liu X., Zhuo S.C., Liu X.Y., Sheng H.H., Sha M., Ye J., Yu H. (2023). A demonstration based on multi-omics transcriptome sequencing data revealed disulfidptosis heterogeneity within the tumor microenvironment of esophageal squamous cell carcinoma. Discov. Oncol..

[23] Qi C., Ma J.M., Sun J.J., Wu X.L., Ding J. (2023). The role of molecular subtypes and immune infiltration characteristics based on disulfidptosis-associated genes in lung adenocarcinoma. Aging.

[24] Wang Y., Tsukamoto Y., Hori M., Iha H. (2024). Disulfidptosis: A Novel Prognostic Criterion and Potential Treatment Strategy for Diffuse Large B-Cell Lymphoma (DLBCL). Int. J. Mol. Sci..

[25] Pavlova N.N., Zhu J.J., Thompson C.B. (2022). The hallmarks of cancer metabolism: Still emerging. Cell Metab..

[26] Liu X.G., Nie L.T., Zhang Y.L., Yan Y.L., Wang C., Colic M., Olszewski K., Horbath A., Chen X., Lei G. (2023). Actin cytoskeleton vulnerability to disulfide stress mediates disulfidptosis. Nat. Cell Biol..

[27] Zheng W., Zheng Y., Bai X., Zhou Y., Yu L., Ji D., Leng K., Meng N., Wang H., Huang Z. (2022). RPNs Levels Are Prognostic and Diagnostic Markers for Hepatocellular Carcinoma. J. Oncol..

[28] Ahn H.R., Baek G.O., Yoon M.G., Son J.A., Yoon J.H., Cheong J.Y., Cho H.J., Kang H.C., Eun J.W., Kim S.S. (2022). Hypomethylation-mediated upregulation of the WASF2 promoter region correlates with poor clinical outcomes in hepatocellular carcinoma. J. Exp. Clin. Cancer Res..

[29] Sauzeau V., Beignet J., Bailly C., Vergoten G. (2022). Overexpressed or hyperactivated Rac1 as a target to treat hepatocellular carcinoma. Pharmacol. Res..

[30] Li T.Z., Yi J.W., Wu H.J., Wang K., Zhou B.H. (2024). SLC7A11 in hepatocellular carcinoma: Potential mechanisms, regulation, and clinical significance. Am. J. Cancer Res..

[31] Quintero-Fabián S., Arreola R., Becerril-Villanueva E., Torres-Romero J.C., Arana-Argáez V., Lara-Riegos J., Ramírez-Camacho M.A., Alvarez-Sánchez M.E. (2019). Role of Matrix Metalloproteinases in Angiogenesis and Cancer. Front. Oncol..

[32] Scheau C., Badarau I.A., Costache R., Caruntu C., Mihai G.L., Didilescu A.C., Constantin C., Neagu M. (2019). The Role of Matrix Metalloproteinases in the Epithelial-Mesenchymal Transition of Hepatocellular Carcinoma. Anal. Cell. Pathol..

[33] Kurilla A., László L., Takács T., Tilajka A., Lukács L., Novák J., Pancsa R., Buday L., Vas V. (2023). Studying the Association of TKS4 and CD2AP Scaffold Proteins and Their Implications in the Partial Epithelial-Mesenchymal Transition (EMT) Process. Int. J. Mol. Sci..

[34] Huang R., Zhang X., Gracia-Sancho J., Xie W.F. (2022). Liver regeneration: Cellular origin and molecular mechanisms. Liver Int..

[35] Jin X., Jin W., Tong L., Zhao J., Zhang L., Lin N. (2024). Therapeutic strategies of targeting non-apoptotic regulated cell death (RCD) with small-molecule compounds in cancer. Acta Pharm. Sin. B.

[36] Pimentel J.M., Zhou J.Y., Wu G.S. (2024). Autophagy and cancer therapy. Cancer Lett..

[37] Tang D., Kroemer G., Kang R. (2024). Targeting cuproplasia and cuproptosis in cancer. Nat. Rev. Clin. Oncol..

[38] Tian Z., Song J., She J., He W., Guo S., Dong B. (2024). Constructing a disulfidptosis-related prognostic signature of hepatocellular carcinoma based on single-cell sequencing and weighted co-expression network analysis. Apoptosis.

[39] Zhu J., Wu Y., Ji P., Qi J., Yang X., Pocha C., Tustumi F., Biachi de Castria T. (2024). Identification of a prognostic disulfidptosis-related gene signature in hepatocellular cancer. J. Gastrointest. Oncol..

[40] Liang J.Y., Wang D.S., Lin H.C., Chen X.X., Yang H., Zheng Y., Li Y.H. (2020). A Novel Ferroptosis-related Gene Signature for Overall Survival Prediction in Patients with Hepatocellular Carcinoma. Int. J. Biol. Sci..

[41] Bushue N., Wan Y.-J.Y. (2010). Retinoid pathway and cancer therapeutics. Adv. Drug Deliv. Rev..

[42] Chen C., Xu J., Zhang J.X., Chen L.Y., Wei Y.A., Zhang W.M., Shao P.F., Xu H.G. (2024). CD2AP is a potential prognostic biomarker of renal clear cell carcinoma. Cancer Med..

[43] Chen X., Zhang Y.W., Ren H., Dai C., Zhang M., Li X., Xu K., Li J., Ju Y., Pan X. (2024). RNF5 exacerbates steatotic HCC by enhancing fatty acid oxidation via the improvement of CPT1A stability. Cancer Lett..

[44] Leahy C., Osborne N., Shirota L., Rote P., Lee Y.K., Song B.J., Yin L., Zhang Y., Garcia V., Hardwick J.P. (2024). The fatty acid omega hydroxylase genes (CYP4 family) in the progression of metabolic dysfunction-associated steatotic liver disease (MASLD): An RNA sequence database analysis and review. Biochem. Pharmacol..

[45] Xie W.K., Chen C., Han Z., Huang J.J., Liu X., Chen H.J., Zhang T.M., Chen S., Chen C.B., Lu M.D. (2020). CD2AP inhibits metastasis in gastric cancer by promoting cellular adhesion and cytoskeleton assembly. Mol. Carcinog..

[46] Zhang L., He J.W., Zhao W.T., Zhou Y.H., Li J., Li S.B., Zhao W.P., Zhang L.L., Tang Z.Q., Tan G.W. (2024). CD2AP promotes the progression of glioblastoma multiforme via TRIM5-mediated NF-kB signaling. Cell Death Dis..

[47] Mirfakhar F.S., Castanheira J., Domingues R., Ramalho J.S., Guimas Almeida C. (2024). The Alzheimer’s Disease Risk Gene CD2AP Functions in Dendritic Spines by Remodeling F-Actin. J. Neurosci..

[48] Shibue T., Weinberg R.A. (2017). EMT, CSCs, and drug resistance: The mechanistic link and clinical implications. Nat. Rev. Clin. Oncol..

