# Supplementary material for: Integrated Analysis of Disulfidptosis-Related Genes Identifies CD2AP as a Potential Therapeutic Target for Hepatocellular Carcinoma
Source: Int J Mol Sci. 2025 May 7;26(9):4454. doi: 10.3390/ijms26094454 (PMC12072785; doi:10.3390/ijms26094454)
Supplement: Supplementary file 1 [file ijms-26-04454-s001.zip › ijms-3558902-supplementary.pdf]

Integrated analysis of disulfidptosis-related genes identifies CD2AP as a potential therapeutic target for hepatocellular carcinoma

Supplementary files

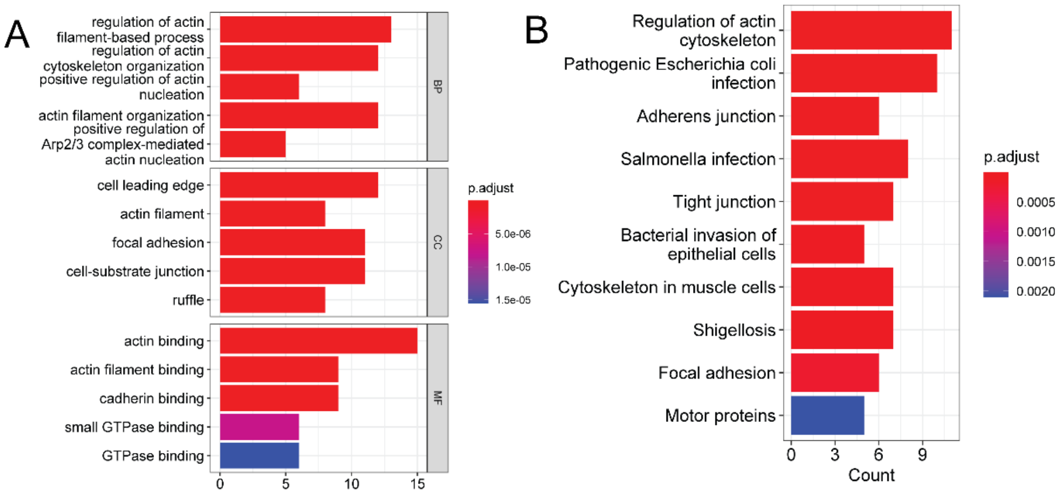

Figure S1. GO and KEGG enrichment of based on 26 disulfidptosis genes. (A) BP, CC and MF category of enrichment. (B) KEGG pathway enrichment analysis.

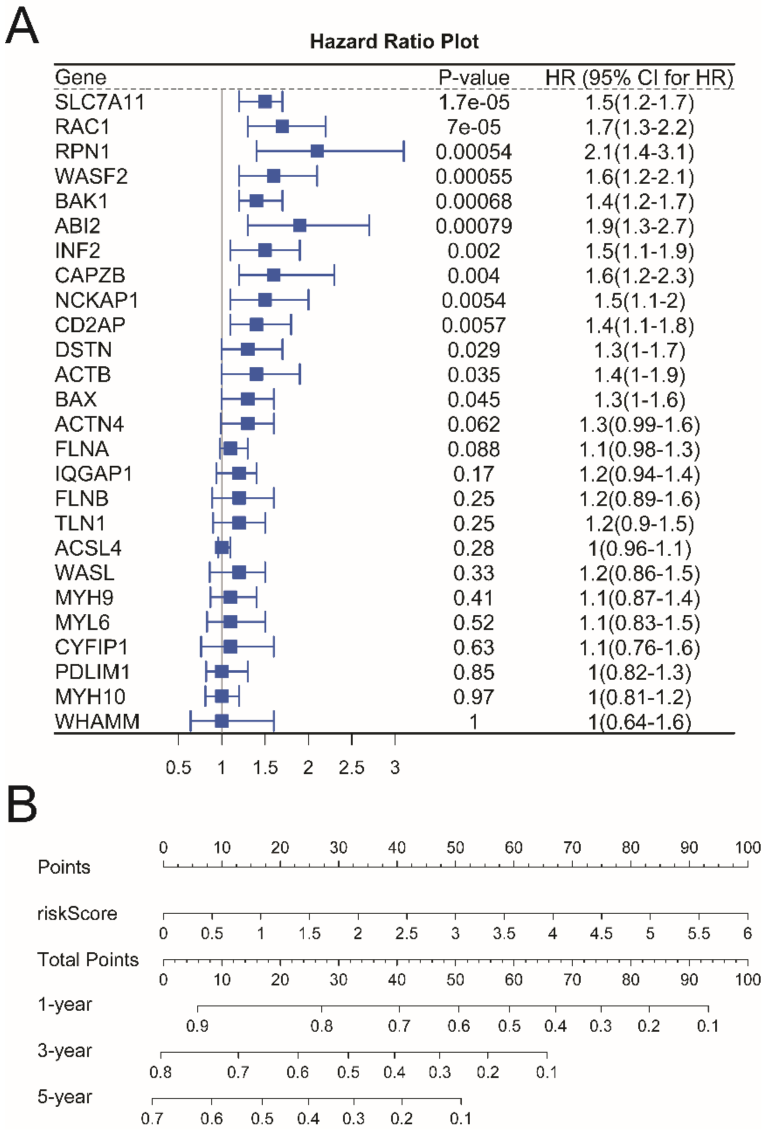

Figure S2. (A) Univariate Cox risk analysis of 26 disulfidptosis genes and (B) Nomogram was constructed to predict the 1-year, 3-year and 5-year OS rates of HCC patients in the TCGA cohort.

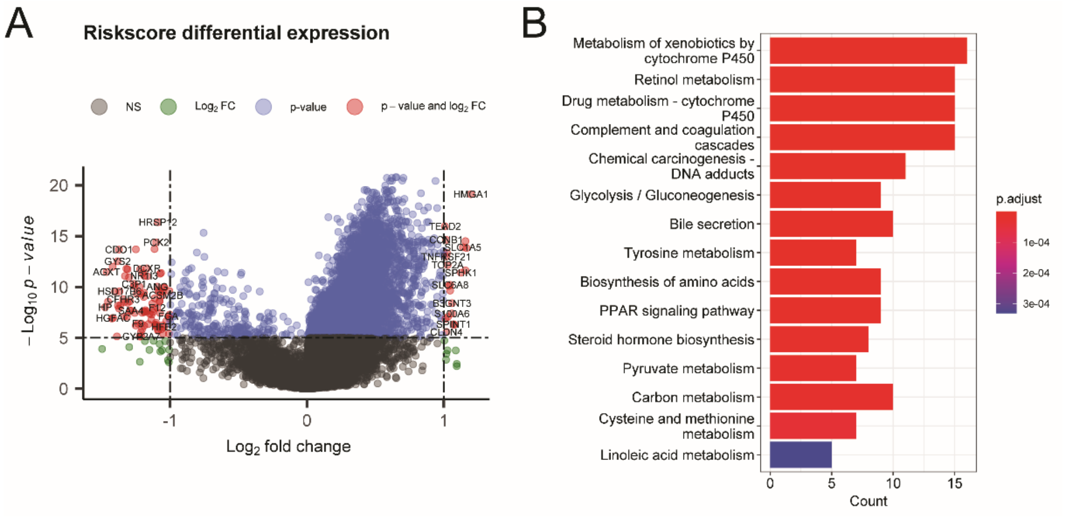

Figure S3. Functional enrichment analyses of differentially expressed genes associated the 7-genes prognostic signature model. (A) Volcano plot of DEGs between high-risk group and low-risk group. (B) KEGG pathway enrichment analysis.

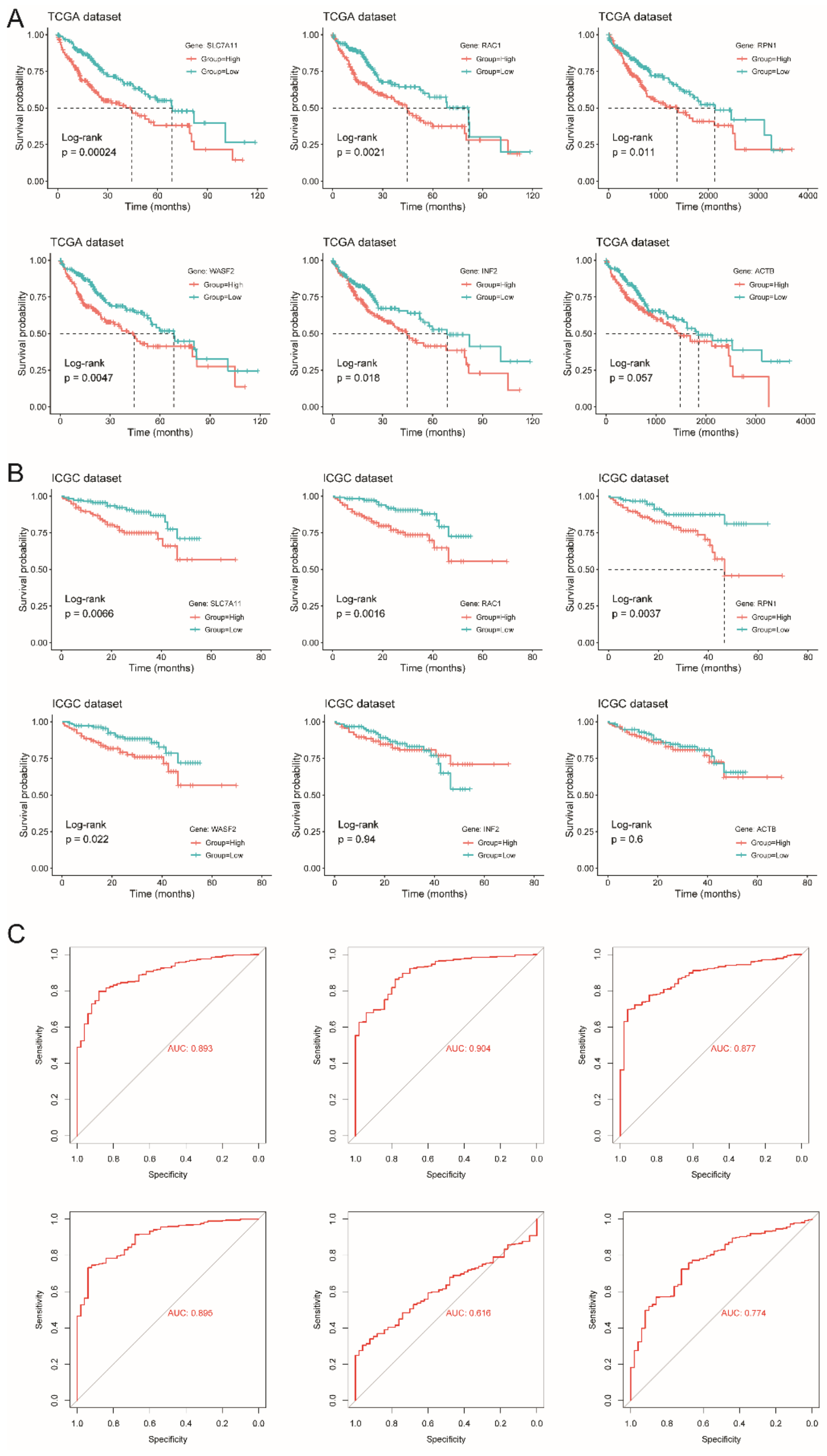

Figure S4. The prognostic signature of the other 6 genes in TCGA and ICGC dataset. The survival plot of the gene expression group in (A) TCGA and (B) ICGC dataset. (C) The ROC curve plots of the genes in TCGA and ICGC dataset.

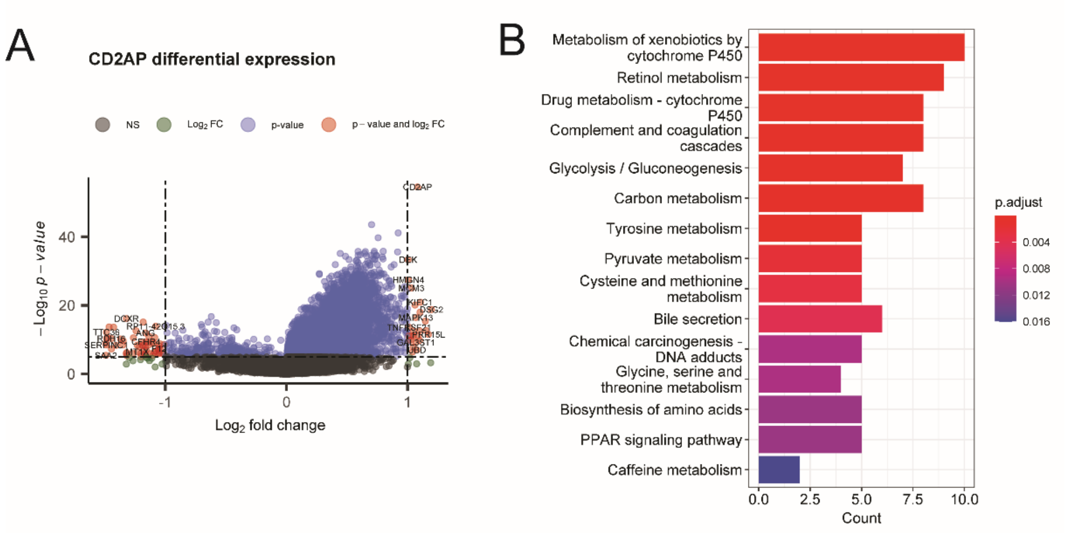

Figure S5. Functional enrichment analyses of differentially expressed genes associated the CD2AP expression group. (A) Volcano plot of DEGs between CD2AP high- and low-expression group. (B) KEGG pathway enrichment analysis.

TableS1. 26 disulfidptosis genes

Num	Gene	Full Name
1	ABI2	abl interactor 2
2	ACSL4	acyl-CoA synthetase long chain family member 4
3	ACTB	actin beta
4	ACTN4	actinin alpha 4
5	BAK1	BCL2 antagonist/killer 1
6	BAX	BCL2 associated X
7	CAPZB	capping actin protein of muscle Z-line subunit beta
8	CD2AP	CD2 associated protein
9	CYFIP1	cytoplasmic FMR1 interacting protein 1
10	DSTN	destrin
11	FLNA	filamin A
12	FLNB	filamin B
13	INF2	inverted formin 2
14	IQGAP1	IQ motif containing GTPase activating protein 1
15	MYH10	myosin heavy chain 10
16	MYH9	myosin heavy chain 9
17	MYL6	myosin light chain 6
18	NCKAP1	NCK associated protein 1
19	PDLIM1	PDZ and LIM domain 1
20	RAC1	Rac family small GTPase 1
21	RPN1	ribophorin I
22	SLC7A11	solute carrier family 7 member 11
23	TLN1	talin 1
24	WASF2	WASP family member 2
25	WASL	WASP like actin nucleation promoting factor
26	WHAMM	WASP homolog associated with actin, golgi membranes and microtubules
